# The Röntgen Society

**Published:** 1897-06-26

**Authors:** 


### The Rontgen Society.

The Röntgen Society is now established, and has held its first general meeting. Professor Silvanus Thompson, the president, delivered the introductory address, in which he said he hoped that the membership of the society would extend to other countries, and that their published transactions might occupy an important place in the literature of science.

